# Exploratory Evaluation of EGFR-Targeted Anti-Tumor Drugs for Lung Cancer Based on Lung-on-a-Chip

**DOI:** 10.3390/bios12080618

**Published:** 2022-08-09

**Authors:** Jianfeng Tan, Xindi Sun, Jianhua Zhang, Huili Li, Jun Kuang, Lulu Xu, Xinghua Gao, Chengbin Zhou

**Affiliations:** 1 Department of Thoracic Surgery, Shenzhen Hospital, Southern Medical University, Shenzhen 518101, China; 2 The Second School of Clinical Medicine, Southern Medical University, Guangzhou 510030, China; 3 Materials Genome Institute, Shanghai University, Shanghai 200444, China; 4 Department of Cardiovascular Surgery, Guangdong Provincial Cardiovascular Institute, Guangdong Provincial People’s Hospital, Guangdong Academy of Medical Sciences, Guangzhou 510030, China

**Keywords:** lung-on-a-chip, drug evaluation, EGFR, lung cancer, targeted therapy

## Abstract

In this study, we used three-dimensional (3D) printing to prepare a template of a microfluidic chip from which a polydimethylsiloxane (PDMS)lung chip was successfully constructed. The upper and lower channels of the chip are separated by a microporous membrane. The upper channel is seeded with lung cancer cells, and the lower channel is seeded with vascular endothelial cells and continuously perfused with cell culture medium. This lung chip can simulate the microenvironment of lung tissue and realize the coculture of two kinds of cells at different levels. We used a two-dimensional (2D) well plate and a 3D lung chip to evaluate the effects of different EGFR-targeting drugs (gefitinib, afatinib, and osimertinib) on tumor cells. The 3D lung chip was superior to the 2D well plate at evaluating the effect of drugs on the NCI-H650, and the results were more consistent with existing clinical data. For primary tumor cells, 3D lung chips have more advantages because they simulate conditions that are more similar to the physiological cell microenvironment. The evaluation of EGFR-targeted drugs on lung chips is of great significance for personalized diagnosis and treatment and pharmacodynamic evaluation.

## 1. Introduction

Lung cancer is the most common cancer worldwide and the most common cause of cancer-related deaths, with more than 2.2 million new cases of lung cancer and 1.8 million deaths from lung cancer being reported in 2020 [1]. Studies have shown that non-small cell lung cancer (NSCLC) accounts for 85% of all lung cancer types; the sensitive mutation rate of adenocarcinoma tissue in patients with advanced NSCLC patients in mainland China is 48.0%, and the overall epidermal growth factor receptor (EGFR) mutation rate is 50.2% [2]. For patients with advanced NSCLC and a positive EGFR gene drive, epidermal growth factor receptor tyrosine kinase inhibitors (EGFR-TKIs) are the current first-line treatment option [3,4,5,6], but these lung cancer patients still have tumor recurrence and aggressive metastasis. The five-year survival rate of non-small cell lung cancer is less than 20% [7]. Moreover, there are individual differences between different lung cancer patients, and there are also differences in their sensitivities to different targeted drugs. To improve the efficacy of EGFR-targeted anti-lung cancer drugs, screening in vitro before clinical treatment is an important step, and such drug screening is valuable to choose an appropriate regimen and individualized treatment for each patient. Therefore, drug screening has become essential for improved drug efficacy and patient prognosis [8,9].

Detection platforms for drug screening mainly include two-dimensional (2D) cell cultures, three-dimensional (3D) organoid cultures, and animal models. In vitro cultures of 2D tumor cell lines or immortalized cell lines is a common method of studying tissue pathophysiology and drug response, but they cannot reproduce the structural, mechanical, and functional properties of human tissues and cannot mimic the inherent complex properties of tissues and organs. Organoids are organ-specific multicellular 3D cultures that reproduce some key structural and functional properties of the corresponding organs [10], but they cannot mimic biophysical properties, thus hindering their application in disease modeling and drug screening. Animals are also used to model human physiology for disease research, preclinical drug development, and screening. However, due to the differences between animals and humans, there are large differences in the physiological structures, tissue and organ functions, life support, etc. Thus, animal models cannot provide a strong theoretical basis for the development and research of human lung models in vitro for disease modeling and drug development and screening [11]. Moreover, animal experimental research has other limitations, such as a long model establishment cycle, high cost, low efficiency, and single research time point; in addition, animal experiments also involve ethical issues. Therefore, it is crucial to develop an intuitive and reliable drug screening detection platform to guide the individualized treatment of lung cancer.

Microfluidic organ chips, with their advantages of high throughput, automation, real-time multi-index monitoring, and accurate simulation of the physiological microenvironment in vivo, can predict the curative effect and toxicity of drugs more quickly and accurately and have great potential in the development of drug screening [12,13,14]. Among them, the lung organ chip is one of the earliest researched organ chips, which can simulate the physiological microenvironment, such as the alveolar structure and respiratory membrane, and has been widely studied and applied [15,16,17,18]. Compared with a conventional cell culture in vitro, a lung organ chip model can simulate lung physiological and pathological conditions more accurately. Lung organ chips can also solve the shortcomings of animal experiments, such as long cycles, high costs, and ethical problems. They are expected to provide a low-cost alternative for studying human organ physiology and organ diseases as well as advancing toxicology research and drug screening [19,20]. Xu Z. et al. used microfluidic chip technology to conduct an experimental study on the drug sensitivity efficacy of individualized chemotherapy drugs and gefitinib in a 3D cell culture model. The results showed that the efficacy of the combined drug group was significantly better than that of the single-agent group [9]. Jain A. et al. used lipopolysaccharide endotoxins (LPS) to infect upper human alveolar epithelial primary cells in a whole blood perfused alveolar chip, which indirectly stimulated endothelial cells in lower 3D blood vessels by activating alveolar epithelial cells, resulting in pulmonary thrombosis [21]. Alveolar chips can also allow for the rapid screening of antithrombotic drugs. Mulholland et al. proposed a microfluidic platform for drug screening of cancer cell-rich multicellular spheroids from tumor biopsies, allowing extensive anticancer drug screening prior to treatment [22]. These studies demonstrate the potential of lung organ chips to facilitate personalized tumor therapy.

**Figure 1:**
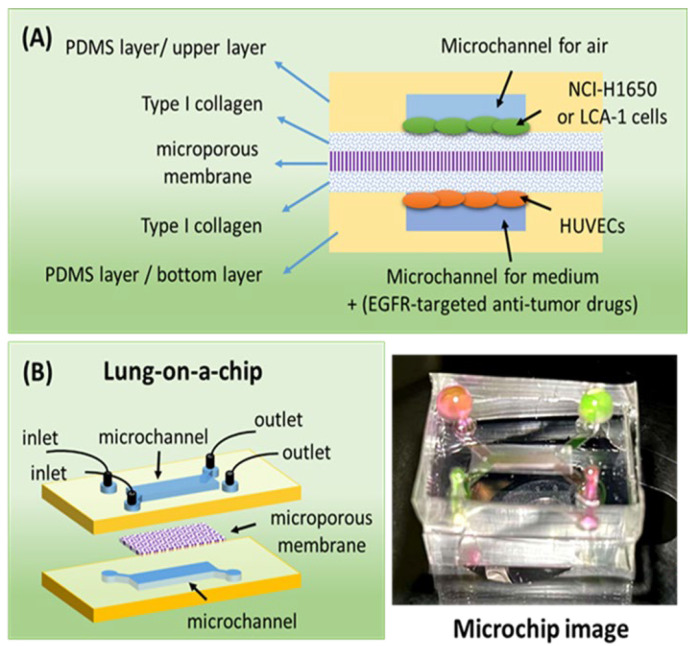
(**A**) A schematic diagram of the experimental principle. (**B**) A schematic diagram and image of the lung-on-a-chip.

At present, polydimethylsiloxane (PDMS) is the main material of lung chips, and lung chips are mainly prepared via soft lithography [23,24,25,26], but the photoresist template preparation steps are cumbersome, requiring glue mixing, baking, exposure, development, etc. When using emerging 3D printing technology to prepare lung chips, the operation is simple, the preparation cost is low, and the chips can be mass-produced. To date, there has been no relevant research using EGFR gene drive-positive primary lung cancer cells to screen EGFR-targeted anti-lung cancer drugs in lung organ chips. In this study, we used 3D printing technology to prepare a resin template of the microfluidic chip from which a PDMS sandwich lung chip was successfully constructed. The upper and lower channels of the chip are separated by a microporous membrane. The upper channel is seeded with lung cancer cells, and the lower channel is seeded with vascular endothelial cells and continuously perfused with a cell culture medium. The schematic design and principle of the chip are shown in Figure 1. The results show that the lung organ chip with a microporous membrane can simulate the microenvironment of lung tissue and realize the coculture of lung cancer cells and vascular endothelial cells at different levels with a high rate of survival. We used two strategies, a 2D well plate and a 3D lung chip, to evaluate the effects of different EGFR-targeting drugs (gefitinib, afatinib, and osimertinib) on NCI-H650 cells and primary lung cancer cells.

## 2. Materials and Methods

### 2.1. 3D Printing Manufacturing of Chip Templates

The chip templates were first prepared by using a 3D printing device (nanoArch^®^ SI40, BMF Material Technology Inc., Shenzhen, China) according to the equipment manual. Identical templates were prepared for the top and bottom layers. First, we used drawing software to design the chip template. We determined that the length, width, and height of the templates should be within the range allowed by the 3D printer (≤9400 μm, ≤5200 μm, and ≤3000 μm, respectively), to design the microchannel and liquid inlet or outlet. Then, we used BMF_3Dslice software to transform the designed 3D model into pictures, adjust the size of the model, assign its location relative to the print platform, and create slices. Finally, we loaded the sliced 3D model into the computer connected to the 3D printer. According to the number of pictures and the required accuracy of the chip templates, we adjusted the descending distance and ascending distance of our platform: the descending distance (M-d) was 4 cm, and the ascending distance (M-u) was 3.97 cm. The descending and ascending waiting times of the platform were as follows: the descending waiting time was 40 s, the ascending waiting time was 60 s, and the exposure time (photosensitive resin curing time) for each descending and ascending step was 7 s. We set the stitching mode to automatic printing, and then the chip template was successfully printed. To ensure the flatness of the upper surface of the chip template and the accuracy of the channel, it was necessary to clean the photosensitive resin remaining between the channels of the chip template with a large amount of ethanol to ventilate and dry at room temperature. The prepared chip template was photographed using an inverted fluorescence microscope (IX-73, Olympus, Tokyo, Japan), and the microchannel heights of the chip template were measured by a stylus profiler (AlphaStep D-300 Stylus Profile, KLA, Milpitas, CA, USA).

### 2.2. Microchip Fabrication

We treated the prepared resin template with silanization; the template could then be used to mold the PDMS layer, and the PDMS could be sealed with a microporous membrane to prepare the microfluidic chip. In detail, we first added 3 μL of 1H,1H,2H,2H- perfluorooctyl triethoxysilane (Sigma, Louis, MO, USA) to the resin template for silanization treatment and placed it in an oven at 80 °C for 6 h. We then mixed PDMS (Sylgard 184, Dow Corning, Midland, MI, USA) and its curing agent at a ratio of 10:1, poured it into the resin template, vacuumed it until the bubbles were removed, and put it in an oven at 80 °C for 1 h to polymerize the PDMS, and removed it from the mold after cooling. The PDMS layer was complete after punching the inlet/outlet holes. We placed the upper PDMS layer with channels face up, cut the polycarbonate microporous membrane to the appropriate size (pore size 5 µm, Neuro Probe), and adjusted it to fit the channels with tweezers. We carried out plasma treatment on the upper PDMS layer and the bottom PDMS layer covering the microporous membrane. After 60 s, we took it out, aligned and sealed it, put it in an oven at 80 °C for 1 h, and then aligned and placed it on the PDMS with tweezers. We plasma treated the top PDMS layer (with membrane) and bottom PDMS layer for 60 s. We then removed the pieces, aligned and sealed them, and then placed the chip in an oven at 80 °C for 1 h. The follow-up experiments were carried out after ultraviolet sterilization.

### 2.3. Diffusion Characterization of Porous Membrane

We used the dye diffusion method to verify the permeability of the microporous membrane in the chip. Rhodamine 123 (RH-123, Sigma, Louis, MO, USA) aqueous solution with a concentration of 10 μg/mL was added to the microchannel of the upper layer of the microchip, and rhodamine B (RH-B, Sigma, Louis, MO, USA) aqueous solution with a concentration of 10 μg/mL was added to the microchannel of the bottom layer of the microchip. Focusing the microscope on the microchannel of the bottom layer, we obtained the changes in the diffusion of RH-123 in the microchannel from the upper layer to the bottom layer by taking time-lapse pictures by confocal microscopy (FV3000, Olympus, Tokyo, Japan).

### 2.4. Isolation of Primary Lung Cancer Cells

This study was approved by the Medical Ethics Committee of Shenzhen Hospital of the Southern Medical University (Ethics number: SZYYEC2021R074). To test the efficiency of this new platform, we also performed a drug evaluation on fresh primary lung cancer cells positive for EGFR Exon19. The patient, a 69-year-old female, did not receive chemotherapy or radiotherapy before the operation. After obtaining informed consent from the patient, lung cancer tissue was obtained during an operation on 5 August 2021. After the operation, the invasive adenocarcinoma was confirmed by pathological examination and analysis. The gene test indicated that the EGFR Exon 19 was positive. According to the UCII (2017) standard, there was one case of stage IA3. Immediately after removal, the tissue was immersed in a culture medium and kept cold during transportation to the laboratory. Necrotic areas, fatty tissue, blood clots, and connective tissue were removed. Then, the tumor was chopped, digested with collagenase I (0.3 mg/mL) at 37 °C for 2 h, collected, and centrifuged (200× *g*, 5 min). Sediments were prepared as single cells suspended in serum-free Dulbecco’s modified Eagle’s medium (DMEM) and separated into different fractions by Percoll discontinuous gradient centrifugation (400× *g*, 20 min; 30% and 70% Percoll). The cells at the interface of 30% and 70% Percoll were collected, and gradient medium was removed with PBS to obtain human primary lung cancer cells (LCA-1) [9].

### 2.5. Cell Culture

Human non-small cell lung cancer NCI-H1650 cells were purchased from the Cell Bank/Stem Cell Bank of the Chinese Academy of Sciences (sourced from ATCC). HUVECs were purchased from BeNa Culture Collection Co., Ltd. (sourced from ATCC, Suzhou, China). NCI-H1650 cells and HUVECs were cultured in Roswell Park Memorial Institute Medium 1640 (1640, Gibco) containing 10% fetal bovine serum (Gibco) and 1% penicillin–streptomycin double antibody (Gibco). Human primary lung cancer cells (LCA-1) were cultured in DMEM (high glucose; Gibco) containing 10% fetal bovine serum (Gibco) and 1% penicillin–streptomycin double antibody (Gibco). The cells were cultured in an incubator at 37 °C and 5% CO_2_.

### 2.6. Cell Staining

The cells were labeled with Cell Tracker according to the manufacturer’s instructions. Cell Tracker Green CMFDA was used to label NCI-H1650 cells, and Cell Tracker Red CMTPX was used to label HUVECs. After coculturing on both sides of the microporous membrane, fluorescence images of the cells were obtained with a laser scanning confocal microscope. In addition, calcein-AM staining was used to evaluate cell viability. The staining process was carried out as reported in our previous research [27].

### 2.7. Data Statistics

ImageJ software and confocal microscope software were used to identify the diffusion distance, cell location, cell morphology, and fluorescence intensity in the fluorescent dye diffusion images and cell staining images.

## 3. Results and Discussion

### 3.1. Evaluation of 3D Printing Chip Templates and Microchip

**Figure 2:**
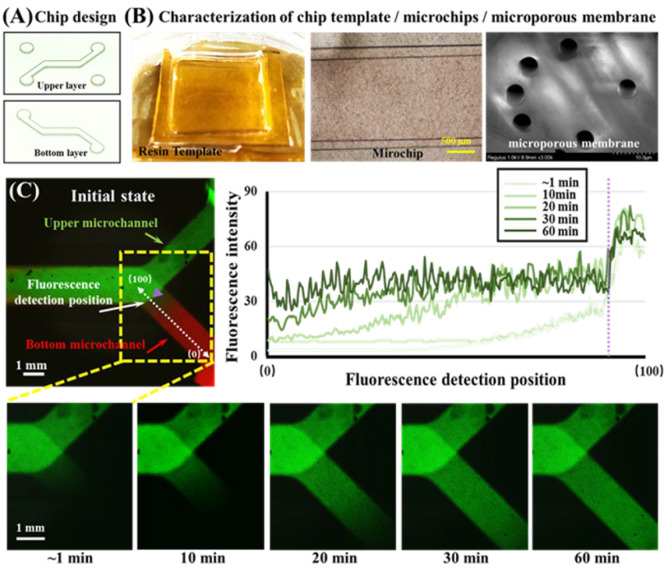
(**A**) Design of the 3D printed chip template. (**B**) Microscopy or SEM images of chip templates, microchips, and microporous membranes. (**C**) Fluorescence images and plots of fluorescence intensity profiles for the characterization of small molecule diffusion in a microchip. Green, RH-123 aqueous solution. Red, RH-B aqueous solution. Scale bar = 1 mm.

In this study, the microchip templates were prepared by 3D printing, including the upper layer template and the bottom layer template. They have a similar structure, as can be seen in Figure 2A. The two templates have the same microchannel structure, with a design height of 1840 μm and a width of 390 μm, which connects the inlet and outlet (each with a diameter of 3.0 mm). To facilitate adding cells and drugs to the microchannel in the bottom layer, another inlet and outlet were added in the upper layer, which was connected to the bottom layer. The prepared chip template is shown in Figure 2B. After measurement, the actual size of the template microchannel was 398 ± 2 μm high and 1846 ± 5 μm wide. The slight deviation from the design is due to the accuracy of 3D printing. However, the error is very small and does not affect the use in this study. Compared with traditional photolithography methods, the 3D printing method simplifies the steps and does not require glue adjustment, baking, exposure, development, etc., which greatly simplifies the labor and time costs. The equipment has also been simplified, eliminating the need for the spin coater, oven, and UV exposure machine, meaning that we only needed a 3D printer. This makes this method more suitable for commercial manufacturing. However, it can also be seen that the precision of 3D printing will affect the precision of the size of the chip template and the smoothness of the surface. The height error of the template we manufactured was about 2 µm, and the channel width error was close to 10 µm. The accuracy of 3D printing is not as good as that of traditional lithography, so this method is more suitable for templates with simple patterns that do not require high channel sizes or heights. In addition, after the upper and lower layers and the microporous membrane were sealed using plasma, we characterized the microchip with a microscope, and it could be seen that the microchannel was clearly defined. We also carried out SEM characterization of the microporous membrane, and the micropore size was relatively uniform and approximately 5 μm. This size can ensure that when cells are seeded, suspended cells (generally about 10 μm in diameter) will not pass through the microporous membrane. Furthermore, during subsequent cell culture or drug stimulation, the molecules can diffuse easily and the cells can be continuously supplied with a culture medium to maintain cell survival. Next, after sealing, photographs of the areas where the upper and lower microchannel meet the membranes will be unclear because the microporous membrane is translucent. Thus, during cell experiments, the cells need to be observed by means of fluorescent staining. Overall, it is feasible to manufacture microchips using 3D printing to prepare resin templates, and the size error of the channel is within an acceptable range. This method is more suitable for large-scale preparation than conventional methods of preparing PDMS templates.

### 3.2. Diffusion Characterization of Small Molecules in Chips

In this study, to simulate the process in which small drug molecules stimulate lung tumor cells in vivo, we applied a fluid containing EGFR-targeting drugs to one side of the channel, which allowed the small drug molecules to enter the other side of the channel through the pores of the microporous membrane by diffusion. To verify the feasibility of small molecule diffusion, we used RH-123 dye molecules with molecules similar to the EGFR-targeted drugs as fluorescent probes, and real-time observation was performed by fluorescence photography. First, the RH-123 and RH-B aqueous solutions were injected into the upper and bottom channels, respectively. The merged fluorescence image is shown in Figure 2C. At the beginning (~1 min), RH-123 (green) and RH-B (red) were in their respective channels due to the presence of the membrane. After that, we tracked the fluorescence changes of the RH-123 dye only. The RH-123 dye was observed on the channel on the other side after approximately 10 min and the channel was completely filled after 30 min. To show the change in fluorescence intensity more intuitively, we define the branch end of the bottom channel as position 0, define the intersection of the upper and bottom channels as position 100, and use the fluorescence intensity change curve at different times to describe the changes in fluorescence intensity of RH-123 from position 0 to position 100. RH-123 can easily pass through the microporous membrane with a pore size of 5 microns, which means that when an EGFR-targeted drug with a similar molecular weight was added to one side channel, it could also easily enter the other channel through the micropore to achieve drug administration.

### 3.3. Stratified Co-Culture of HUVEC and NCI-H1650 Cells

**Figure 3:**
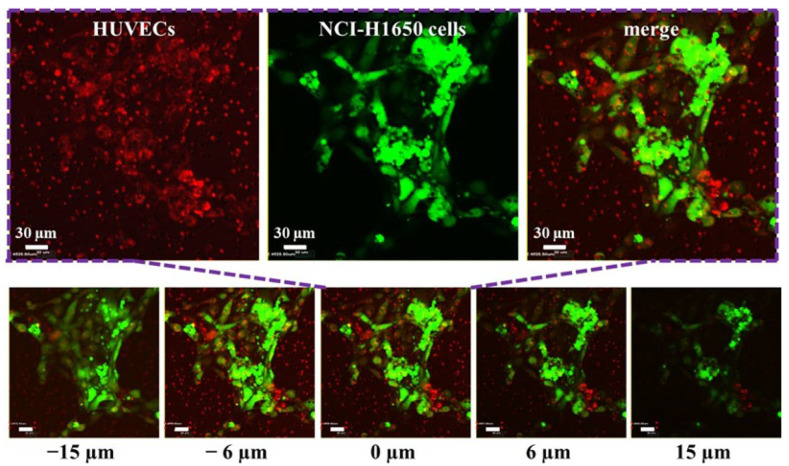
Fluorescence images of HUVECs and NCI-H1650 cells stratified co-cultured on both sides of the microporous membrane. HUVECs (red) were labeled by Cell Tracker Red CMTPX and NCI-H1650 cells (green) were labeled by Cell Tracker Green CMFDA. Scale bars = 30 µm. We defined the z-axis position of the microporous membrane as 0 µm. The indicated distances are measured from the membrane plane (0 µm). The positive values are above it, while the negatives are below.

On the established microchip, we cocultured HUVECs and non-small-cell lung cancer NCI-H1650 cells. In the study, Cell Tracker Red CMTPX and Cell Tracker Green CMFDA dyes were used to label HUVECs and NCI-H1650 cells, respectively. These two dyes that are commonly used in organ-on-a-chip studies are live cell tracking dyes that do not affect cell viability and adhesion properties. To promote the attachment of HUVECs to the microporous membrane, we modified the sterilized microchip with type I collagen. We injected 40 µL of 0.01% (*w*/*v*) type I collagen solution into the microchip from the bottom channel inlet, incubated it at 37 °C for 30 min, and then washed it with PBS buffer solution. Then, 40 µL of the 2 × 10^5^/mL HUVEC cell suspension labeled with Cell Tracker Red CMTPX was added to the bottom channel. Then, the chip was placed upside down in a Petri dish with culture medium and cultured in an incubator for 1 day to wait for HUVECs to adhere to the microporous membrane. Next, 40 µL of the 2 × 10^5^/mL NCI-H1650 cell suspension labeled with Cell Tracker Green CMFDA was added to the upper channel, and the chip was placed in the incubator for 1 day to allow the NCI-H1650 cells to adhere to the microporous membrane. The liquid in the upper channel was carefully removed to fill the channel with air. Then, the precision syringe pump pipes were connected with the inlet of the bottom microchannel. After that, the HUVECs and NCI-H1650 cells were co-cultured for 1 day with continuous medium flow (100 µL/h) controlled by the syringe pump. The HUVECs and NCI-H1650 cells on both sides of the microporous membrane were photographed by confocal microscopy. The results are shown in Figure 3. We defined the z-axis position of the microporous membrane as 0 µm. As can be observed by confocal microscopy, the HUVECs and NCI-H1650 cells adhered to both sides of the microporous membrane. This indicates that the noncontact stratified coculture of HUVECs and NCI-H1650 cells was successfully established by adding cells separately to make them adhere to both sides of the microporous membrane, leading to a structure similar to that of the lung respiratory membrane in vivo. This model is also frequently used to simulate the alveolar or lung bronchial cell microenvironment in vitro in lung-on-a-chip studies.

### 3.4. Evaluation of EGFR-Targeted Drugs for NCI-H1650 Cell

EGFR-TKIs are the current first-line treatment option for patients with advanced NSCLC with a positive EGFR gene drive. Common EGFR-targeting drugs include first-generation ones such as gefitinib, which inhibit the EGFR protein function of cells through competitive binding with ATP and mainly target exon 19 deletion and exon 21-point mutation. The second-generation EGFR-targeting drugs, represented by afatinib, are different from the first-generation drugs. In addition to reversibly binding ATP binding sites on EGFR in a competitive way, they can also be alkylated or covalently (irreversibly) bound with EGFR-specific amino acid residues, mainly targeting exon 19 deletion and exon 21-point mutation. The third-generation EGFR-targeting drugs, represented by osimertinib, can not only competitively and irreversibly bind to the ATP-binding site on EGFR but can also target the most common drug resistance mutation, T790M. In addition to the deletion of exon 19 and the point mutation of exon 21, it can also mutate T790M in exon 20. In view of the clinical efficacy and safety of gefitinib, afatinib, and osimertinib, we chose to evaluate these EGFR-targeted drugs.

**Figure 4:**
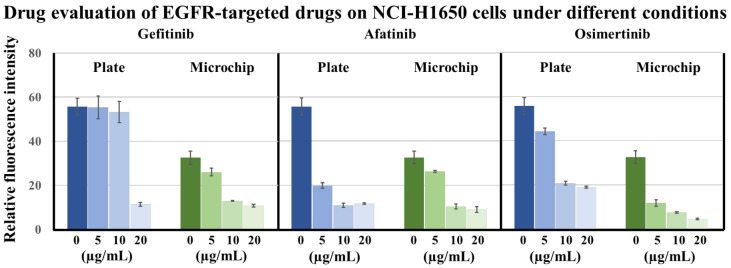
Drug evaluation of EGFR-targeted drugs. Effects of different concentrations of gefitinib, afatinib, and osimertinib on HCI-H1650 cells using a 2D well plate and 3D lung-on-a-chip methods. *n* = 3.

In this study, we used a 2D well plate and a 3D lung chip to test the effects of three kinds of EGFR-targeted drugs, (i.e., the first-generation EGFR-targeted drug gefitinib, the second-generation EGFR-targeted drug afatinib, and the third-generation EGFR-targeted drug osimertinib) with different concentrations on the viability of HCI-H1650 cells. The three EGFR-targeted drugs at concentrations of 0, 5, 10, and 20 μg/mL were applied to NCI-H1650 cells in well plates or lung chips for 24 h, and calcein-AM staining was used to characterize cell activity. The fluorescence images obtained by confocal microscopy are shown in Supplementary Material Figure S1, and the results were statistically analyzed (Figure 4). In both the 2D well plate and the 3D lung chip, the death rate of HCI-H1650 cells without EGFR-targeted drugs was less than 5%, which can be used as a control group for cell experiments. The average fluorescence intensity in the plate control group (0 μg/mL) was generally higher than that of the microchip control group (0 μg/mL). This is mainly because, under the same magnification, the cells observed in fluorescence images of the microchip group are only those that have adhered and survived in the microchannel, so the cell adhesion area was less than that in the plate group, and the value after calculating the average intensity was lower. Regarding the effects of different concentrations of targeted drugs on HCI-H1650 cell activity, the first-generation drug gefitinib significantly reduced the cell activity when it was 20 μg/mL compared with the well plate control group. Afatinib and osimertinib at lower concentrations of 5 μg/mL or 10 μg/mL reduced the activity of HCI-H1650. This showed that the second-generation and third-generation drugs were superior to the first-generation drugs in the treatment of pulmonary non-small-cell lung cancer, which was similar to the results reported in the literature [4,5]. On the other hand, compared with the chip control group, gefitinib and afatinib greater than 10 μg/mL and osimertinib greater than 5 μg/mL could significantly reduce cell activity. In particular, the third-generation targeted drug osimertinib showed better efficacy on the lung chip. At the same concentration, osimertinib was more effective than afatinib and gefitinib. This was consistent with the current clinical results. Clinical studies have shown that the median progression-free survival with the third-generation targeted drug osimertinib is approximately 18.9 months, which is better than the median progression-free survival with the second-generation targeted drug afatinib (approximately 11 months) and that of the first-generation targeted drug gefitinib (approximately 10 months). The third-generation drug is better than the second-generation drug, and the second-generation drug is better than the first-generation drug [4,5,6]. The above showed that for the drug evaluation of EGFR-targeted drugs in NCI-H1650 cells, the 3D lung chip method was better than the 2D culture method, which showed the feasibility of our prepared 3D lung chip for the evaluation of EGFR-targeted drugs in vitro.

### 3.5. Evaluation of EGFR-Targeted Drugs for Primary Lung Cancer Cells

**Figure 5:**
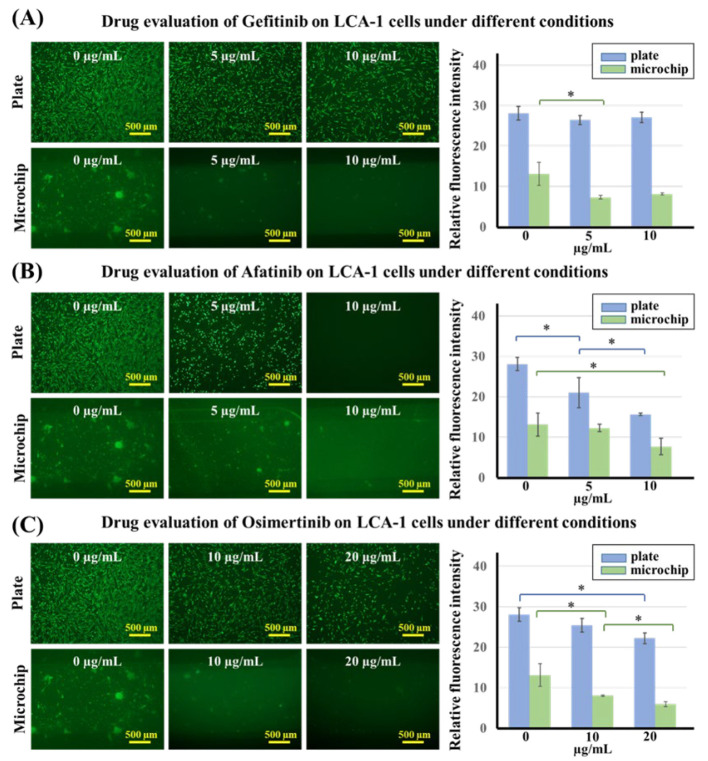
(**A**) Drug evaluation of EGFR-targeted drugs with different concentrations of gefitinib on LCA-1 cells using the 2D well plate and 3D lung-on-a-chip methods. (**B**) Drug evaluation of EGFR-targeted drugs with different concentrations of afatinib on LCA-1 cells using the 2D well plate and 3D lung-on-a-chip methods. (**C**) Drug evaluation of EGFR-targeted drugs with different concentrations of osimertinib on LCA-1 cells using the 2D well plate and 3D lung-on-a-chip methods. *n* = 3, * *p* < 0.05.

We also used 2D well plates and 3D lung chips to test the effects of the three kinds of EGFR-targeted drugs, gefitinib, afatinib, and osimertinib, at different concentrations on the viability of LCA-1 primary lung cancer cells. The LAC-1 primary lung cancer cells were determined to be positive for EGFR Exon 19 after genetic testing, and EGFR-targeted drugs should thus have an effect on it. The first-generation EGFR-targeted drug gefitinib was used at three concentrations (0, 5, and 10 μg/mL), which were tested on LCA-1 cells in 2D well plates and 3D lung chips. The fluorescence images were obtained by confocal microscopy, and the results were statistically analyzed as shown in Figure 5A. The results showed that gefitinib had no significant effect on cell activity in the 2D well plate group, and the cells showed a certain level of drug resistance. However, gefitinib partially reduced cell activity in the 3D lung chip group. With the second-generation EGFR-targeted drug afatinib (results shown in Figure 5B) in the 2D well plate group, cell viability gradually weakened as the concentration increased, suggesting that the drug concentration is positively correlated with cell death; however, 5 μg/mL afatinib in the lung chip was not enough to induce significant cell death. In addition, osimertinib (results shown in Figure 5C) had no significant effect on cells at 5 μg/mL, so the drug concentration was adjusted to 0, 10, and 20 μg/mL, and it was found that the cell activity decreased gradually as the concentration increased in the 2D well plate group and decreased significantly at 20 μg/mL. In the 3D lung chip group, osimertinib also affected cell viability in a concentration-dependent manner. Furthermore, for the same drug concentration of 10 μg/mL, only afatinib was effective on cells in the 2D well plate, while cells were evidently not sensitive to that concentration of gefitinib or osimertinib. On the other hand, in the 3D lung chip group, each of the three drugs at a concentration of 10 μg/mL had an effect on cell viability. The sensitivity of primary lung cancer cells to the three kinds of EGFR-targeted drugs was not as typical as that of lung cancer cell lines in both 2D plates and 3D chips, which may be due to the heterogeneity of primary tumor cells. However, compared with the 2D well plate, the 3D lung chip puts forward higher requirements for primary cell culture on the one hand, and on the other hand, it more realistically simulates the microenvironment of lung tumor cells in vivo by including the gas environment, blood flow stimulation, and cell co-culture. These factors in the chip are very likely to change the cell’s morphology and sensitivity to drugs. In the presence of cell co-culture and fluid factors, primary lung cancer cells in the microchip were more likely to grow into clumps compared with a 2D plate or NCI-1650 cells, which was similar to their behavior in vivo. Overall, we believe that the 3D lung chip can more closely simulate the real tumor environment when it comes to drug efficacy evaluation, as well as a series of other factors, such as topographic structure, cocultured endothelial cells, fluid flow, and gas environment, which were similar to the real environment in vivo, indicating the feasibility of the organ-chip method.

## 4. Conclusions

We developed a microporous membrane lung chip based on 3D printing manufacturing technology, which realizes the coculture and fluid delivery strategy of lung cancer cells and vascular endothelial cells at different levels. The effects of different EGFR-targeted drugs (gefitinib, afatinib, and osimertinib) on NCI-H650 cells and primary lung cancer cells were tested in 2D well plates and 3D lung chips. The results show that the 3D lung chip is better than the 2D well plate for the evaluation of the effects of the drugs on the non-small cell lung cancer NCI-H650 cell line, and the data from the microchip have more advantages, as they can more closely match the existing clinical drug data. For primary tumor cells, the 3D lung chip can provide conditions that are more in line with the physiological cell microenvironment. The evaluation of EGFR-targeted drugs on the lung chip is of great significance to personalized diagnosis and treatment and pharmacodynamic evaluation.

## Data Availability

The data that support the findings of this study are available from the corresponding author upon reasonable request.

## Supplementary Materials

The following supporting information can be downloaded at: https://www.mdpi.com/article/10.3390/bios12080618/s1, Figure S1: Fluorescence images of LCA-1 cells treated with different concentrations of gefitinib, afatinib, and osimertinib for 24 h. Scale bar = 500 μm.
