# Supplementary material for: Exploratory Evaluation of EGFR-Targeted Anti-Tumor Drugs for Lung Cancer Based on Lung-on-a-Chip
Source: Biosensors (Basel). 2022 Aug 9;12(8):618. doi: 10.3390/bios12080618 (PMC9405841; doi:10.3390/bios12080618)
Supplement: Supplementary file 1 [file biosensors-12-00618-s001.zip › biosensors-1822918-supplementary.pdf]

Supplementary Material

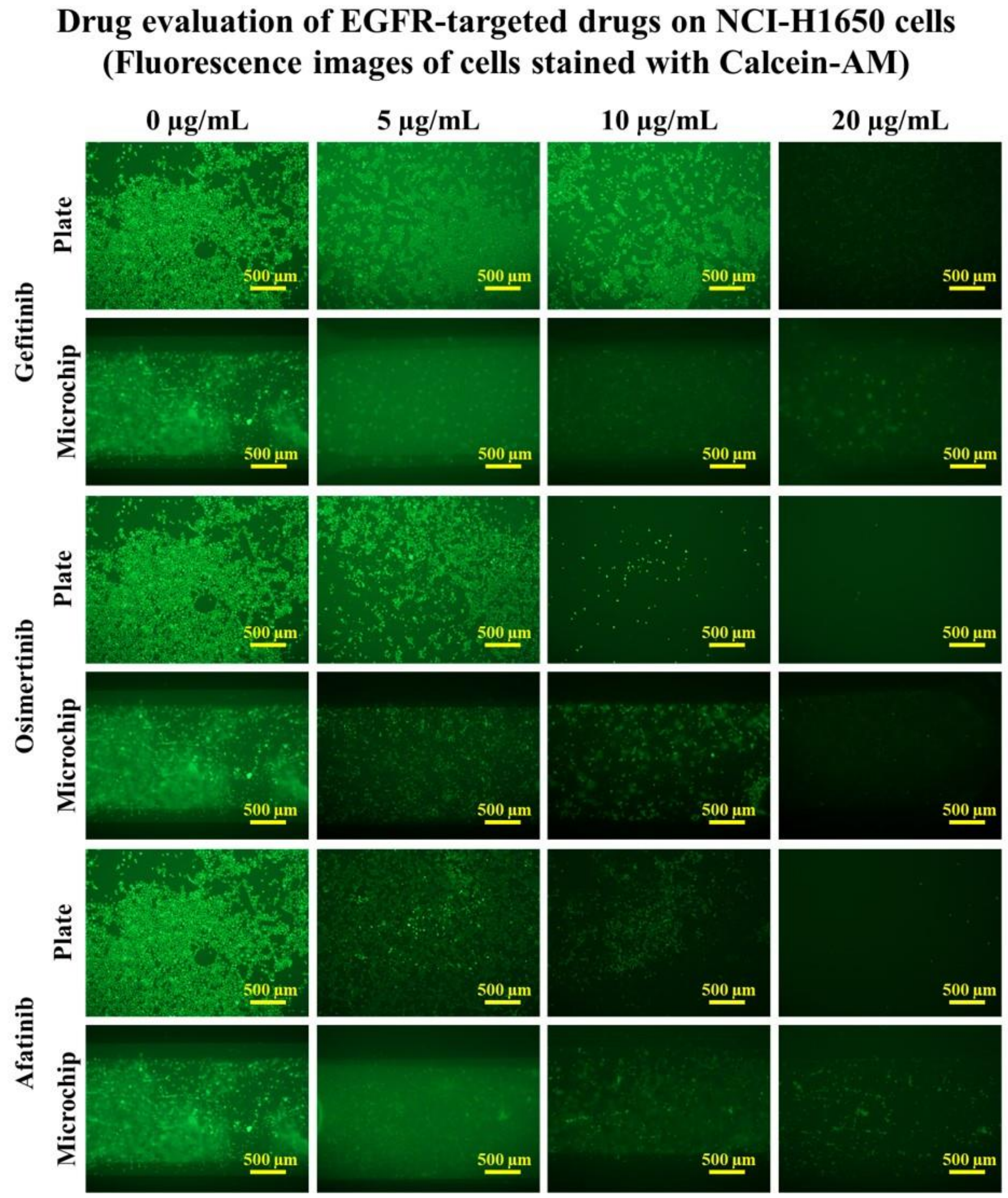

Figure S1. Fluorescence images of LCA-1 cells treated with different concentrations of gefitinib, afatinib, and osimertinib for 24 h. Scale bar = 500 μm.
